# Bone events and evolution of biologic markers in Gaucher disease before and during treatment

**DOI:** 10.1186/ar3111

**Published:** 2010-08-09

**Authors:** Jérôme Stirnemann, Nadia Belmatoug, Corine Vincent, Olivier Fain, Bruno Fantin, France Mentré

**Affiliations:** 1 Médecine Interne, Hôpital Jean-Verdier, Assistance Publique-Hôpitaux de Paris, Université Paris XIII, Referral Center for Lysosomal Diseases (RCLD), Avenue du 14 juillet, 93140 Bondy Cedex, France; 2 INSERM, UMR 738, Université Paris-Diderot, Hôpital Bichat, 46, rue Henri-Huchard, 75877 Paris Cedex 18, France; 3 Médecine Interne, Hôpital Beaujon, Assistance Publique-Hôpitaux de Paris, Université Paris VII, Referral Center for Lysosomal Diseases (RCLD), 100, boulevard du Général Leclerc, 92110 Clichy Cedex, France

## Abstract

**Introduction:**

Known biomarkers of Gaucher-disease activity are platelets, chitotriosidase, angiotensin-converting enzyme (ACE), tartrate-resistant acid phosphatase (TRAP) and ferritin. The aim of this study was to retrospectively evaluate the frequency of bone events (BE) and biomarker changes during two periods: diagnosis to first enzyme-replacement therapy (ERT) and the latter to the closing date.

**Methods:**

BE of 62 treated patients, among the 73-patient cohort followed at Beaujon Hospital, Clichy, France, were described with Kaplan-Meier curves, and linear-mixed models were used to analyze their biomarker changes and the influence of several covariates (splenectomy, diagnosis year, genotype, age at diagnosis and sex).

**Results:**

BE occurred before (54 events in 21 patients), but also during, ERT (12 events in 10 patients), with respective frequencies (95% confidence interval) at 10 years of 22.4% (13.3 to 36.3) and 20.0% (10.2 to 36.9). Biomarker slope changes before and during ERT differed significantly for platelets (+190/mm^3^/year and 7,035/mm^3^/year, respectively; *P *< 0.0001) and ferritin (+4% and -14%; *P *< 0.0001). High ferritin levels and low platelet counts at ERT onset were significantly associated with BE during ERT (*P *= 0.019 and 0.039, respectively). Covariates significantly influenced biomarker changes (baseline and/or slope): splenectomy affected platelets (baseline and changes), TRAP changes and chitotriosidase changes; diagnosis date influenced ACE and TRAP baseline values; and genotype influenced chitotriosidase baseline and changes.

**Conclusions:**

Platelet counts and ferritin levels and their slope changes at ERT onset seem to predict BE during treatment. Biomarker baseline values and changes are dependent on several covariables.

## Introduction

Gaucher disease (GD), a rare autosomal-recessive disorder with an approximate prevalence of 1/75,000 live births worldwide, is due to the deficiency of a lysosomal enzyme (glucocerebrosidase, glucosylceramidase or β-glucosidase acid (EC 3.2.1.45)) [1] or, rarely, its activator (saposin C) [2,3]. This lysosomal storage disease is characterized by liver and spleen enlargement, and severe bone complications [1]. Based on the neurological signs, three clinical phenotypes are recognized: type 1, the classic form, affects 95% of the patients and is usually defined by the absence of central nervous system impairment; types 2 and 3 are rare and severe, due to neurological involvement [4].

Type 1 GD has bone complications that can alter the functional prognosis: abnormal bone deformity, such as widening of the femur metaphysis (Erlenmeyer flask), osteopenia, osteoporosis, lytic lesions and pathological and vertebral compression fractures. Bone infarcts are manifested by acute painful bone crises, and avascular necroses lead to degenerative arthropathy that may require replacement by prosthesis [5]. Thrombopenia and anemia are common. Liver enzymes may be slightly elevated and cholestasis may be present. GD diagnosis is confirmed by the detection of low glucocerebrosidase activity, usually less than 30% of the normal value in peripheral leukocytes. Genotyping can sometimes provide prognosis information [6].

Enzyme-replacement therapy (ERT), alglucerase then imiglucerase, available since 1991, is the reference treatment. Substrate-reduction therapy (miglustat) has been available since 2002, and is indicated for moderate GD when ERT is unsuitable. These treatments are extremely expensive. ERT appears to be ineffective against the onset of neurological disorders in type 2 [4,7]. To our knowledge, no complete analysis of bone complications occurring under ERT is available. Bone complications generally decline after two years of ERT [8]. However, no data are available on the main bone events (BE; avascular necrosis, bone infarcts, pathological fractures) occurring during ERT.

Several biomarkers (chitotriosidase, ferritin, angiotensin-converting enzyme (ACE) and tartrate-resistant acid phosphatase (TRAP)) are elevated during GD evolution [9-16]. Their concentrations rise with disease progression and generally decrease during ERT [17]. At present, it is not possible to make any formal recommendations concerning the use of any specific marker for patient monitoring [18]. Moreover, it is not known if biomarker levels at diagnosis can predict GD prognosis of treated and untreated patients, and which patients will respond, or not, to therapy [19]. Despite the lack of official guidelines, ACE, TRAP and chitotriosidase are used to monitor GD follow-up [20].

Several studies on chronic diseases used biomarker modeling to describe their evolution: human immunodeficiency virus infection [21], Parkinson disease [22] and diabetes mellitus [23]. Concerning GD patients, most published studies are descriptions of small cohorts (median number of patients, 29; range, 18 to 48) [9,10,17,19,24,25] and only one study modeled hemoglobin and platelet levels and splenic volume under ERT [26].

Therefore, this study was undertaken to analyze BE frequencies occurring during the periods before and during ERT in our cohort of GD patients and to model the progression of their biological marker levels or slope changes.

## Materials and methods

### Patients and data collected

The Referral Center for Lysosomal Diseases (RCLD) is specialized in GD follow-up. A designated French national GD registry was developed and is maintained by the RCLD. Although patients are treated and followed in hospitals near their homes, they are registered with the RCLD, which is available to assist their physicians. However, a cohort of patients is followed and treated on-site in the RCLD. All patients with known GD entered in the RCLD registry, followed on-site in the Department of Internal Medicine, Beaujon Hospital (Clichy, France), and receiving ERT were included. Clinical, biological and radiological data were recorded for all patients from diagnosis until 1 May 2007, the closing date. Data were collected retrospectively for two periods: before and during ERT.

Written consent was obtained from each patient. The local Institutional Review Board of Northern Paris Hospitals, Paris-Diderot University, AP-HP (Ethics Committee) reviewed and approved the research project.

A standardized case-report form was used to collect the following information at each visit: initial data (age, sex, history related or unrelated to GD, initial symptoms and their year of onset, test confirming the diagnosis, phenotype, genotype, unknown genetic mutation); clinical information during the first consultation, at diagnosis and throughout follow-up; organomegaly (liver and/or spleen), usually measured using diagnostic ultrasonography (largest diameter); biological findings initially and throughout follow-up. Bone findings (X-rays, magnetic resonance imaging and, for some patients, scintigraphy and dual-energy X-ray absorptiometry) were recorded during follow-up, with identification of intercurrent events, particularly bone complications.

BE were defined as clinical events using the bone indications for treatment recommended by the French National Health Authority [27]: avascular necrosis of an epiphysis, bone infarct, pathological and/or vertebral compression fracture(s). Each BE had a clinical manifestation and radiological confirmation. Bone pain alone was not considered a BE without radiological confirmation.

Monitoring of GD-specific ERT and combined therapies (analgesics and bisphosphonates) were noted.

GD diagnosis was confirmed by low glucocerebrosidase activity in leukocytes [28] for all patients. Chitotriosidase activity in plasma samples was determined using the fluorescent substrate 4-methyl umbeliferyl β-*d-N*,*N*',*N*"-(MU)-triacylchitotriose [9]; ACE, TRAP, ferritin and other measurements were made in the appropriate local laboratories. Because this study was retrospective, some data were missing, particularly at the beginning of follow-up (during the diagnosis phase). When missing, the baseline value at ERT onset was replaced with the last known value during the two previous years. When the chitotriosidase concentration was undetectable (patients with homozygous chitotriosidase-gene deficiency), this biomarker was not retested [29].

### Statistical analysis

All statistical analyses were performed with SAS software (version 9.1; SAS Institute Inc, Cary, North Carolina, USA). The significance level was set at *P *< 0.05.

First, we described BE frequency using Kaplan-Meier probability-of-BE curves to determine the time to the first BE for treated patients between diagnosis and their first treatment (before ERT), and between first ERT and the closing date (during ERT). Only the first BE occurring during each period was considered. Data were censored if no BE occurred before the patient started ERT for the first analysis and until the closing date for the second analysis. The LIFETEST procedure was used.

Second, we analyzed the changes of the five GD biomarkers (platelets, chitotriosidase, ferritin, ECA, TRAP) using linear-mixed models for repeated measures with the MIXED procedure. Because of their minimal variations during ERT, hemoglobin levels were not included in this model. The MIXED procedure is a generalization of a standard linear regression, which allows modeling of the parameter changes for each individual over time and takes into account the intrasubject association. Biomarker changes over time could have one of two shapes: either a linear increase or an exponential decrease. For the latter, logarithmic transformation was used in the model. Models of platelet changes used only the counts of nonsplenectomized patients. Two categories were created and analyzed: before and during ERT, regardless of the dose, with analysis of patients receiving full-dose ERT as a subcategory. Before-and-during ERT slopes were compared using the Wald test.

Third, we analyzed the effects of five covariates on BE: splenectomy, diagnosis year (before 1991 or after 1991, the year ERT became available), genotype (*N370S*/*N370S *or others), age at diagnosis (before 15 or after 15 years old) and sex. The impact of each covariate on the time to the first BE was tested using the log-rank test. A Cox model, used to estimate hazard ratios (HR) and 95% confidence intervals (CI), was applied to patients before and during ERT. Influence of age at treatment onset on BE occurrence under ERT was tested with a Cox model.

Fourth, the covariates were tested in mixed models. These analyses were only applied to the patients under ERT because of insufficient data on the patients before ERT. Backward selection of the covariates entered into the model was applied to examine associations between a biomarker and the different covariates. For the before-ERT and during-ERT analysis periods, individual baseline and slope values estimated with the linear-mixed models with no covariate for each patient and for each biomarker were extracted. These values were entered into a Cox model, to evaluate the relationship between BE and biomarker changes using the PHREG procedure, and are expressed as HR and 95% CI.

A biomarker effect on BE occurrence before ERT was not analyzed because early information on biomarkers was very sparse.

## Results

### Cohort

Seventy-three patients were followed, between 1933 and 1 May 2007, for a median duration of follow-up of 21 (range, 0 to 67) years after diagnosis. Only 62 patients received ERT with a median total duration of follow-up from diagnosis of 23.5 (range, 2 to 67) years and median duration of follow-up under treatment of 6 (range, 0 to 15) years. Only these 62 patients were included in the analysis.

The patients' characteristics at diagnosis are reported in Table 1. This mostly female cohort had a median age of 14 years at diagnosis, but their first symptoms had started at the median age of eight years. Bone-marrow aspiration or biopsy led to GD diagnosis for 32 (51%) patients, and spleen histology was used for 13%. The diagnosis was confirmed for all patients by determining glucocerebrosidase activity. Only one patient died of GD-associated pulmonary hypertension during follow-up.

**Table 1:** Description at diagnosis of the 62 Gaucher-disease patients receiving enzyme-replacement therapy

Baseline characteristic	Value
Sex, *n (%)*	
Female	36 (58)
Male	26 (42)
Age, years, *median (range)*	
First symptoms	8 (0 to 37)
Diagnosis	14 (1 to 48)
Patients diagnosed before 1991, *n (%)*	47 (76%)
Patients <15 years old at diagnosis, *n (%)*	34 (55%)
First symptoms to diagnosis interval, years, *median (range)*	1 (0 to 36)
Test leading to diagnosis, *n (%)*	
Enzyme assay	7 (11)
Enzyme-gene sequencing	1 (2)
Myelogram	26 (42)
Bone-marrow biopsy	4 (6)
Bone biopsy	2 (3)
Hepatic biopsy	2 (3)
Spleen histology	8 (13)
Other	1(2)
Unknown	11 (18)
Phenotype, *n (%)*	
1	58 (93)
3	4 (7)
Genotype, *n (%)*	
*N370S*/*N370S*	9 (14)
*N370S*/*L444P*	12 (19)
Other	27 (44)
Unknown	14 (23)
Familial disorder, *n (%)*	
Yes	28 (45)
No	23 (37)
Unknown	11 (18)

All but four patients had phenotype 1 GD. Twenty-eight patients had familial GD affecting siblings for 27 patients and an uncle for one patient. The genotype was known for more than 90% of the patients, including nine *N370S*/*N370S*, 12 *N370S*/*L444P *and two *L444P*/*L444P*.

The first ERT prescribed was alglucerase for 18 (29%) patients and imiglucerase for 44 (71%); ERT was started at a median of 14 (range, 0 to 61) years after diagnosis; median age (range) at ERT onset was 31.6 (4.4 to 65.9) years. The respective median ages (range) at treatment onset for patient with and without BE were 29.5 (15.6 to 51.1) years and 32 (4.4 to 65.9) years. Cox analysis results showed no influence of age at treatment onset on BE occurrence during ERT (HR = 1.017 (95% CI: 0.975 to 1.061), *P *= 0.42). All patients taking alglucerase were switched to imiglucerase in November 1996. For alglucerase or imiglucerase, the initial dose was 120 U/kg/month (full dose) for 55 patients, with lower doses for the others: median 90 (range, 30 to 90) U/kg/month. Four patients switched from imiglucerase to miglustat. Twenty-eight of the 55 patients receiving full doses had their doses lowered after a median of 2.9 (range, 0.1 to 12.2) years of ERT.

Table 2 shows clinical, biological and bone data at different times: diagnosis, ERT onset and closing date. However, median times to ERT onset differed when GD had been diagnosed before 1991 or after 1991 (respectively 18 and 7 years; *P *< 0.05). Median ages at ERT onset and the closing date were 33.3 and 39.8 years, respectively.

**Table 2:** Clinical, biological and imaging characteristics of Gaucher disease precisely known at each time

Characteristic	**No**.	At diagnosis	**No**.	At ERT onset	**No**.	At closing date
Years since diagnosis, *median (range)*		0		14 (0 to 61)		23.5 (2 to 67)
Clinical involvement						
Pigmentation	35	6%	35	20%	29	3%
Asthenia	45	42%	52	60%	53	26%
Abdominal pain	45	29%	50	34%	54	6%
Chronic bone pain	1	0%	52	58%	55	45%
Bone crisis	38	24%	45	49%	51	12%
Hemorrhage	44	52%	53	43%	53	9%
Lung	41	2%	49	0%	52	6%
Neurological	40	5%	45	7%	41	15%
Other	28	4%	32	6%	19	0%
Splenectomy, *n*	62	5	62	21	62	21
Organomegaly						
Hepatomegaly	48	85%	47	89%	39	54%
Liver US *(median, range)*, cm	23	16.5 (13 to 25)	28	19 (13 to 30)	9	14 (11.8 to 19)
Splenomegaly	40	100%	41	95%	30	67%
Splenic US *(median, range)*, cm	34	18.75 (9.5 to 30)	26	19.4 (9.5 to 31.5)	11	15.2 (9 to 22)
Biological parameter, *median (range)*						
Hemoglobin (g/dL)	15	11.4 (7.9 to 14.1)	55	12 (8.3 to 15.1)	58	13.8 (7.3 to 16.2)
Leukocyte (/mm^3^)	15	4,300 (2,070 to 12,400)	54	4,200 (1,180 to 21,600)	57	6,130 (850 to 11,600)
Platelet count (×10^3^/mm^3^)	57	88 (6 to 380)	53	87 (30 to 449)	58	165.5 (37 to 473)
Chitotriosidase (nmol/mL/h)*	28	9,501 (70 to 77,500)	27	9,700 (180 to 77,500)	53	1123 (8 to 14,893)
TRAP (IU/L)	23	11 (1 to 47)	15	9.6 (1 to 24.5)	36	3.75 (2 to 48)
ACE (IU/L)	28	259.5 (1 to 650)	21	220 (1 to 650)	46	51 (0.9 to 240)
Ferritin (ng/L)	38	682.5 (68 to 3,230)	28	721.5 (120 to 3,230)	47	167 (15 to 1,731)
Gammaglobulin (g/L)	2	17.7 (16.5 to 19)	31	15.8 (7.2 to 25)	47	12.7 (6.5 to 23.6)
Imaging of bone disorders						
Erlenmeyer flask	23	52%	22	64%	14	36%
Osteopenia	23	57%	18	56%	17	47%
Cortical	19	32%	13	23%	9	0
Lytic lesion	19	26%	14	21%	15	33%
Avascular necrosis sequelae	27	37%	17	29%	13	23%
Infarct sequelae	22	32%	15	40%	12	25%
Fracture sequelae	18	17%	11	0	11	27%
Infiltration on MRI	25	80%	33	91%	31	81%
^99m^Tc-Hyperfixation	25	84%	29	90%	25	88%
^99m^Tc-Hypofixation	4	50%	5	20%	3	0
Bone densitometry, *median (range)*						
T-score neck	3	-2.1 (-2.2 to -1.1)	10	-1 (-2.2 to 1.4)	22	0 (-2.8 to 4.5)
T-score lumbar	13	0 (-3.1 to 1)	10	-1.9 (-4 to 0.8)	22	-0.9 (-3.6 to 1.6)
Z-score neck	7	8 (-2.03 to 8)	7	-0.7 (-2 to 1.9)	15	-0.3 (-2.6 to 4.4)
Z-score lumbar	2	-2.4 (-3.1 to -1.8)	8	-1.3 (-3.1 to 0.5)	15	-0.5 (-3 to 2.1)

No. is the number of patients with available information. ACE, angiotensin-converting enzyme; ERT, enzyme-replacement therapy; MRI, magnetic resonance imaging; TRAP, tartrate-resistant acid phosphatase; US, ultrasonography. *In addition, four patients had undetectable chitotriosidase activity (null allele) and are not included for statistical analysis of chitotriosidase.

During ERT, clinical abnormality rates decreased (except for neurological involvement) and the biological data improved overall during ERT (increased hemoglobin, leukocyte and platelet levels; decreased chitotriosidase, ACE, TRAP, ferritin and gammaglobulin levels). The number of patients with splenomegaly and/or hepatomegaly tended to decline during ERT, and most bone lesions other than BE tended to regress (Table 2).

Overall, 21 (34%) patients were splenectomized: 5 before diagnosis, 16 between diagnosis and ERT onset; none were splenectomized after starting ERT. The median age at splenectomy was 18.3 (range, 1.6 to 49.6) years.

### BE characteristics

**Figure 1:**
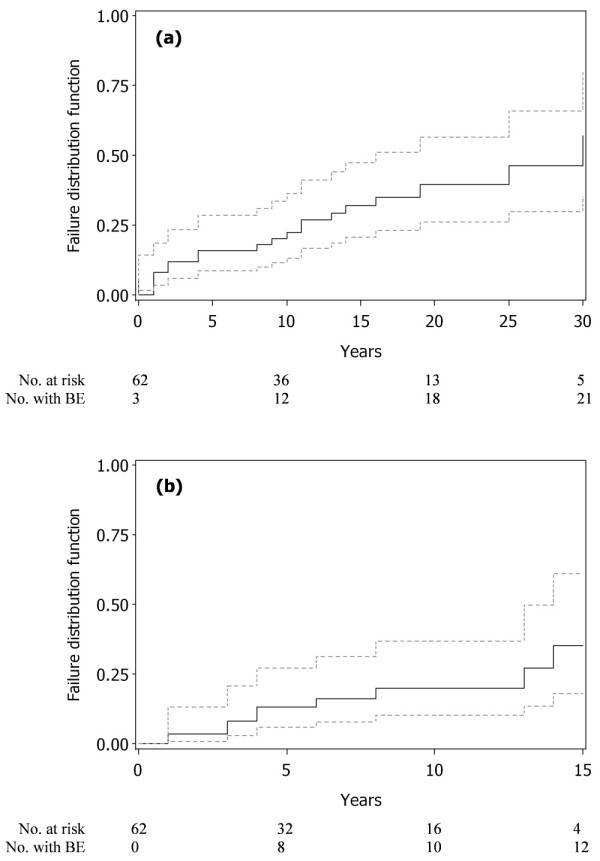
**Time until the first bone event (BE) in the 62 Gaucher-disease patients receiving enzyme-replacement therapy (ERT)**. The dashed grey lines represent the 95% CI of the survival curve. **(a) **Between diagnosis and first ERT during the first 30 years of follow-up. **(b) **Between first ERT and closing date (for treated patients) during 15 years of follow-up. No. at risk is the number of patient followed at the indicated time; No. with BE is the number of patients who had a BE. Twenty-one patients had BE before ERT and 10 under ERT during each follow-up period.

Kaplan-Meier curves of the time to the first BE in the 62 treated patients, between diagnosis and ERT onset (30 years of follow-up), and between the latter and the closing date (15 years of follow-up) are shown in Figure 1a and 1b, respectively. Before diagnosis, eight patients had already suffered at least one BE. After diagnosis, but before starting ERT, 21 patients had had at least one BE, for a total of 54 BE and a median of two (range, 1 to 8) BE per patient. Ten patients had at least one BE during ERT for a total of 12 BE. The 54 BE before ERT onset were (n (%)): 28 (52%) avascular necroses (with 12 prosthetic replacements), 7 (13%) bone infarcts (with only symptomatic therapy), 12 (22%) pathologic fractures (5 requiring surgical intervention) and 7 (13%) vertebral compression fractures (with symptomatic therapy). Moreover, 23 complaints of bone pain were not corroborated by imaging (hence not included in BE). The 12 BE that occurred under ERT were (n (%)): three (25%) avascular necroses (none with prosthetic replacement), four (33%) bone infarcts with clinical bone crises (with only symptomatic therapy) and five (42%) pathological fractures (none requiring surgery). Twenty-one complaints of simple bone pain without imaging confirmation during ERT were not included in BE. For nine of the 12 BE, patients received full-dose ERT (120 U/kg/month). Only one patient experienced BE during the first year of ERT (pathological fracture), and 5 of the 10 patients experienced BE between Years 1 and 5 of ERT (two pathological fractures, one avascular necrosis and two bone infarcts).

We determined the probability of a BE occurring by 10 years (95% CI) before and during ERT: 22.4% (13.3% to 36.3%) and 20.0% (10.2% to 36.9%), respectively. Respective mean times (95% CI) to the first BE were 27.6 (21.5 to 33.7) and 12.0 (10.7 to 13.3) years. For four of the 21 (19.0%) patients with at least one BE before ERT, the BE occurred before and during ERT, whereas six of the remaining 41 (14.6%) patients developed BE only during ERT, but had never done so before.

### Biomarker evolution before and during ERT

Results of analyses of biomarker changes using linear-mixed models are reported in Table 3. Platelet counts in nonsplenectomized patients were stable before ERT (+190 platelets/year), while chitotriosidase and TRAP decreased slightly, and ferritin and ACE increased slightly. During ERT, platelet counts increased (+7,035 platelets/year), while all other biomarkers declined. Slopes before and during ERT differed significantly (*P *= 0.0001) only for platelets and ferritin. For patients given full-dose ERT, platelet counts increased slightly faster, ferritin and TRAP decreased faster, but chitotriosidase and ACE declined more slowly. When only patients with full-dose ERT were analyzed, their biomarker-slope variations were comparable to those of the other patients.

**Table 3:** Changes of the slopes* of the Gaucher-disease biomarkers

	Slope of variation (Unit)						***P***^***a***^
Biomarker	**No./no**.	Before ERT	**No./no**.	During ERT	**No./no**.	Full-dose ERT	
Platelets (no splenectomy)^b^	9/70	190/mm^3^/y	38/480	7,035/mm^3^/y	33/220	10,231/mm^3^/y	<0.0001
Chitotriosidase	10/23	-11%/y	52/226	-17%/y	37/134	-14%/y	0.14
TRAP	7/14	-0.7%/y	36/214	-4%/y	24/109	-6%/y	0.78
ACE	9/33	0.1%/y	46/263	-5%/y	31/114	-4%/y	0.88
Ferritin	9/45	4%/y	47/338	-14%/y	37/166	-16%/y	<0.0001

*Slopes were estimated by linear-mixed models before enzyme-replacement therapy (ERT), during ERT and for the patient subgroup receiving full-dose ERT. ^a^Comparison of slope values before vs during ERT. ^b^Only for nonsplenectomized patients. No./no. indicates number of patients with available data/total number of data available. ACE, angiotensin-converting enzyme; TRAP, tartrate-resistant acid phosphatase.

### Impact of covariates and biomarkers on developing BE

Covariate (splenectomy, date of diagnosis, genotype, age at diagnosis and sex) impact on BE before and during ERT was examined. Before ERT, significantly more BE occurred in patients diagnosed with GD after 15 years of age (HR, 2.6 (95% CI, 1.0 to 6.7); *P *= 0.048), but no significant differences were found for the other covariates, and none had an effect during ERT.

Estimated individual slopes of the biomarkers had no significant influence on developing BE before or during ERT but, at diagnosis, ferritin concentration (HR, 1.18 (95% CI, 1.03 to 1.35); *P *= 0.019) and platelet count (HR, 0.69 (95% CI, 0.49 to 0.98); *P *= 0.039) increased the risk of BE during ERT in this univariate model. Risk of BE increased with high ferritin levels and low platelet levels.

For patients under ERT, a multivariate regression model including age at diagnosis and ferritin and platelet levels found only the baseline platelet count (*P *= 0.032) to have a significant impact.

### Influence of covariates on biomarker changes during ERT

Table 4 reports the effects of covariates on biomarker values during ERT, including the coefficients of variation of interindividual variability for baseline levels and slopes for each biomarker. These coefficients were particularly high for the platelet count, ferritin and ACE slopes. However, only the ferritin and platelet slopes differed significantly between before and during ERT (Table 3).

**Table 4:** Baseline levels and slope changes* of the biomarkers during enzyme-replacement therapy: impact of covariates

Biomarker	Covariate(s)	N	Baseline	*P*	**CV**^**a**^	**Slope of % decrease**^**b**^	*P*	**CV**^**a**^
Platelets								
	No splenectomy	38	108,850 (/mm^3^)		56.5%	7 035 (/mm^3^/yr)		80.7%
	Splenectomy	20	309,380 (/mm^3^)			-882 (/mm^3^/yr)		
	Effect of splenectomy			<0.0001			0.0007	
Chitotriosidase								ND
	No splenectomy-*N370S*	6	14,721 nmol/mL/h		9.8%	-38% (/yr)		
	No splenectomy-other genotype	22	3,581 nmol/mL/h			-14% (/yr)		
	Splenectomy-*N370S*	2	8,283 nmol/mL/h			-27% (/yr)		
	Splenectomy-other genotype	11	2,015 nmol/mL/h			-2% (/yr)		
	Effect of splenectomy			0.19			0.015	
	Effect of genotype			0.006			0.018	
Ferritin		47	382 ng/L		15.4%	-14% (/yr)		83.6%
ACE								
	Diagnosis before 1991	35	90.3 (IU/L)		25.5%	-7% (/yr)		190.1%
	Diagnosis in 1991 and after	11	31.7 (IU/L)			4% (/yr)		
	Effect of diagnosis data			0.020			0.055	
TRAP								
	No splenectomy-diagnosis >15 yr	10	4.2 (IU/L)		3.6%	-4% (/yr)		ND
	No splenectomy-diagnosis 15 yr	13	6.6 (IU/L)			-9% (/yr)		
	Splenectomy-diagnosis >15 yr	5	3.5 (IU/L)			3% (/yr)		
	Splenectomy-diagnosis <15 yr	8	5.4 (IU/L)			-2% (/yr)		
	Effect of splenectomy			0.32			0.004	
	Effect of age at diagnosis			0.016			0.057	

*Estimated with linear-mixed models under treatment. ^a^Coefficient of variation of interpatient variability. ^b^When a log-linear model was used, the evolution is expressed as the percent change of the slope (all markers except platelets). ND, not determined.

**Figure 2:**
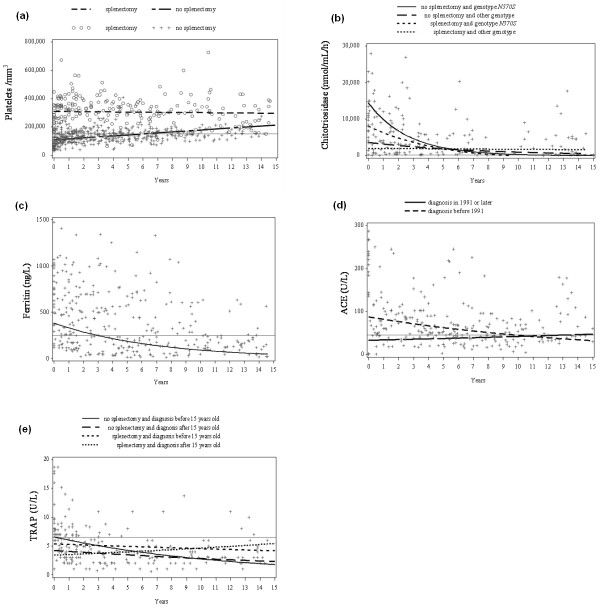
**Biomarker changes under enzyme-replacement therapy (ERT): impact of covariates**. **(a) **platelet level, **(b) **chitotriosidase, **(c) **ferritin, **(d) **angiotensin-converting enzyme (ACE), and **(e) **tartrate-resistant acid phosphatase (TRAP). Different covariates were tested to identify those impacting on biomarker evolution (baseline or variation slope): splenectomy, diagnosis year (before 1991 or after 1991, the year ERT became available), genotype (*N370S*/*N370S *or others), age at diagnosis (before 15 or after 15 years old) and sex. Solid grey horizontal lines correspond to the normal biomarker value: platelet level >150,000/mm^3^; chitotriosidase <100 nmol/mL/h; ferritin <250 ng/L; ACE <45 IU/L; TRAP <7 IU/L. When a covariate had a significant impact on the baseline value or the variation slope, the different curves are shown.

Figure 2 illustrates the influence of covariates on biomarker changes under ERT. Figure 2a shows the influence of splenectomy on the platelet change. Values at diagnosis and progression slopes differed between splenectomized and nonsplenectomized groups. Splenectomized patients' platelet counts rose slightly under ERT, with no significant slope change, while nonsplenectomized patients' counts increased significantly from baseline under ERT but returned to normal after 6.5 years of treatment. Two covariates affected the chitotriosidase decrease under ERT: splenectomy influenced the slope decline and genotype influenced the baseline level (Figure 2b). Patients with the genotype *N370S*/*N370S *had significantly higher baseline levels, which decreased more steeply than those of patients with another genotype. This impact could reflect the fact that, patients with that genotype, which corresponds to one of the less severe forms of GD, started ERT later (median, 23 years) than the other patients (median, 16 years) (nonsiginficant, NS). Chitotriosidase levels decreased, without reaching a normal level over a median of six (range, 0 to 15) years of follow-up.

The ferritin model did not identify any covariate as significantly influencing this marker's progression (Figure 2c). The mean prediction curve for ferritin levels decreased and became the normal after three years.

Date of diagnosis significantly affected baseline ACE levels (Figure 2d). Patients diagnosed before 1991 had much higher ACE levels (90.3 versus 31.7 U/L), but their progression slopes did not differ significantly.

Splenectomy and age at diagnosis affected TRAP levels, with the former influencing the decrease rate (slope) and the latter the baseline concentration (Figure 2e). TRAP levels in splenectomized patients decreased more slowly and had started higher, but the difference was not significant. Patients diagnosed before the age of 15 years had the highest baseline TRAP levels but their diagnosis-to-ERT interval was 17 years, as opposed to 11.5 years for those diagnosed after 15 years (NS). Although that difference was not significant, more substrate could have accumulated in the patients who were diagnosed before the age of 15, because their time preceding ERT onset had been much longer.

Splenectomy eliminates platelet anomalies and displaces lysosomal overload. Nonsplenectomized patients seemed to have higher, but not significantly different, lysosomal biomarker levels at diagnosis compared to splenectomized patients, respectively: chitotriosidase 19,219 versus 2,534 nmol/mL/h (NS); TRAP 12.1 versus 8.0 (NS). In contrast, macrophage-biomarker levels were higher in splenectomized than nonsplenectomized patients, respectively: ferritin 1,301 versus 634 ng/L (*P *= 0.06); ACE 251 versus 202 IU/L (NS). Generally, for splenectomized patients, when the starting value was lower, the slope was less steep.

Some covariates were significantly associated: patients diagnosed before 1991 were more often male (*P *= 0.04) and had more frequently been splenectomized (*P *= 0.03).

## Discussion

Probability curves of time to BE (Figure 1) before and during ERT for GD patients showed that bone complications could occur without but also under ERT (about 20% at 10 years). According to the literature, bone crises almost disappeared after two years of ERT [8,30], but the risk of avascular necrosis was estimated to be 13.8/1,000 person-years under ERT in a recent publication [31]. Our findings demonstrated that BE can arise even after many years of ERT, specifically avascular necrosis (three patients) and bone infarcts (four patients). However, no patient had prosthetic replacement after ERT compared to 12 before. BE after ERT seemed to be less serious, as no surgery was required. In addition, simple bone pain occurring under ERT without being confirmed by imaging (21 events) was not considered a BE. Hence, ERT does not eliminate all bone symptoms. Nine BE were documented under full-dose ERT (120 U/kg/month of alglucerase or imiglucerase). We tried to determine the impact of diagnosis year (before or after 1991) on BE occurrence and biomarker evolution: no effect was found on BE occurrence (*P *= 0.11 and 0.42, respectively, for before and after ERT). Although this covariate significantly affected only baseline ACE levels (see Figure 2d), it had no influence on the other biomarkers, for example, platelets and ferritin. No impact of the date of diagnosis on baseline ferritin value or platelet count was found, as shown in Figure 2a, c and in Table 4.

We hypothesize that bone infiltration accumulating over the years before starting ERT could explain bone complications despite treatment. Unfortunately, our analysis does not allow us to confirm or refute that postulate. In a recent article, the risk of avascular necrosis while on ERT seemed lower for patients who had begun treatment within two years of diagnosis compared to those who started it after more than and equal to two years [31]. Moreover, patients were certainly more closely monitored during than before ERT and it is possible that some BE before ERT might not have been diagnosed. A largest study with systematic bone-density data will be considered to compare the bone densitometries of patients with pathological fractures versus those of patients without such fractures during evolution.

Because the platelet level did not rise in splenectomized patients on ERT, it can be concluded that hypersplenism is the main cause of thrombopenia. However, 14 (61%) out of 21 splenectomized patients, had at least one platelet count <150,000/mm^3 ^after splenectomy. Therefore, bone-marrow insufficiency seems to explain part of the thrombopenia observed over time.

The effect of individual estimated-biomarker values at diagnosis or their slope on BE occurrence under ERT was significant for baseline platelet and ferritin levels in our univariate model, with high ferritin and low platelets at ERT onset being significantly associated with BE during ERT, but only the baseline platelet count was retained in a multivariate model. Nor did the estimated individual slopes of the biomarkers have a significant impact on BE. However, our modeling method seemed to be able to identify predictive biomarkers. Effects of others biomarkers was not significant in our analyses but platelet and ferritin data were available for more patients at diagnosis (57 and 38, respectively) compared to the others (28, 23 and 28, for chitotriosidase, TRAP and ACE, respectively), which could have decreased the power of statistical analysis and partially explain these observations. According to the literature, no biomarkers were able to predict BE occurrence and, other than platelets, ferritin seemed to be the only biomarker affecting BE [19]. The only study using a mixed model for GD [26] found dose-response relationships for ERT, but had not considered biomarkers (chitotriosidase, TRAP, ferritin, ACE), taking into account only the main hematological (hemoglobin and platelets) and visceral manifestations; no prediction of BE occurrence was proposed.

Interpatient biomarker changes and therapeutic responses varied widely (Table 3). TRAP baseline was higher for the group diagnosed before 15 years than those diagnosed later, which seems to support that the TRAP level could differ as a function of age [32]. The respective median ages for the groups of patients diagnosed before and after 15 years were 21 and 40 years, which partially explains these findings. The ACE level was affected by the year of diagnosis, with patients diagnosed after 1991 having lower baseline levels than those diagnosed before 1991. ERT availability since 1991 could have limited the ACE rise. For some biomarkers, not all patients had the same evolution under ERT: for example, platelet counts rose in 97% of treated patients, while chitotriosidase decreased in 100% and ferritin in 96%, but ACE levels in only 78%.

## Conclusions

BE can occur in GD even after many years of ERT. Initial ferritin and platelet levels seemed to be able to predict BE occurrence during ERT. To achieve our final objective, to predict BE based on initial biomarker values and their evolution, large cohort studies are needed.

## Abbreviations

ACE: angiotensin converting enzyme; BE: bone events; CI: confidence interval; ERT: enzyme replacement therapy; GD: Gaucher disease; HR: hazard ratio; NS: nonsignificant; RCLD: Referral Center for Lysosomal Diseases; TRAP: tartrate-resistant acid phosphatase.

## Competing interests

Paris-Diderot University received a grant from Genzyme France. JS and NB were reimbursed for congress expenses by Genzyme. Beaujon Hospital received a grant from Shire.

## Authors' contributions

FM, JS, CV, NB, BF and OF designed the research protocol. JS, NB, BF and OF were involved in treating patients and collecting data. JS, CV and FM controlled the accuracy of collected data and conducted the statistical analyses. JS, FM and CV wrote the draft of the paper, which was then corrected and approved by all authors.
